# Hyperbaric exposure in rodents with non-invasive imaging assessment of decompression bubbles: A scoping review protocol

**DOI:** 10.1371/journal.pone.0274241

**Published:** 2022-09-09

**Authors:** Joshua Currens, Paul A. Dayton, Peter Buzzacott, Virginie Papadopoulou

**Affiliations:** 1 Joint Department of Biomedical Engineering, The University of North Carolina and North Carolina State University, Chapel Hill, North Carolina, United States of America; 2 Curtin School of Nursing, Curtin University, Perth, Australia; Ottawa Hospital Research Institute, CANADA

## Abstract

Hyperbaric pressure experiments have provided researchers with valuable insights into the effects of pressure changes, using various species as subjects. Notably, extensive work has been done to observe rodents subjected to hyperbaric pressure, with differing imaging modalities used as an analytical tool. Decompression puts subjects at a greater risk for injury, which often justifies conducting such experiments using animal models. Therefore, it is important to provide a broad view of previously utilized methods for decompression research to describe imaging tools available for researchers to conduct rodent decompression experiments, to prevent duplicate experimentation, and to identify significant gaps in the literature for future researchers. Through a scoping review of published literature, we will provide an overview of decompression bubble information collected from rodent experiments using various non-invasive methods of ultrasound for decompression bubble assessment. This review will adhere to methods outlined by the Joanna Briggs Institute Manual for Evidence Synthesis and be reported according to the Preferred Reporting Items for Systematic Reviews and Meta-Analyses for Scoping Reviews (PRISMA-ScR). Literature will be obtained from the PubMed, Embase, and Scopus databases. Extracted sources will first be sorted to a list for inclusion based on title and abstract. Two independent researchers will then conduct full-text screening to further refine included papers to those relevant to the scope. The final review manuscript will cover methods, data, and findings for each included publication relevant to non-invasive in vivo bubble imaging.

## Introduction

Decompression sickness (DCS) is a concern for all scuba divers and may occur despite following accepted diving guidelines. Decompression sickness occurs when the ambient pressure surrounding a person is reduced, for example, after exceeding the recommended ascent rate from depth to the surface while diving. Relevant terms to the decompression sickness research field, along with definitions and abbreviations, are listed in Table 1. At depth, scuba divers are subjected to a higher ambient pressure due to the surrounding water. While underwater, divers rely on a breathing apparatus that delivers gas at ambient pressure, increasing the concentration of inert gas molecules within a diver’s bloodstream and tissues [1]. When returning to the surface, the pressure gradient reverses and ambient pressure decreases, causing tissues to become supersaturated with gas that needs to be released into the circulation and eliminated through the lungs, if ample time is allowed [2]. It is suspected that gas bubbles may form in any tissue within the body; however, free flowing bubbles are typically identified within the blood vessels.

**Table 1:** Key terms and their definitions in the context of decompression research.

Key Terms	Definition
Decompression Illness (DCI)	Blanket term encompassing two diseases that can occur after a reduction in ambient pressure, decompression sickness (DCS), and arterial gas embolism (AGE).
Decompression sickness (DCS)	Pathophysiology resulting from the formation and growth of inert gas bubbles during or after a decompression and encompassing a range of possible symptoms.
Arterial gas embolism (AGE)	In the context of scuba diving, AGE is most often the result of pulmonary barotrauma when gas enters the arterial circulation due to accidental breath hold upon ascent resulting in lung overexpansion injury.
Decompression bubble	Any bubble that grows inside the body after a reduction in ambient pressure (decompression). These are presumed to form and grow as a result of inert gas supersaturation in tissues, resulting in bubbles in situ (“tissue bubbles”) or in the circulation (venous side as tissues degas, although bubbles may paradoxically enter the arterial circulation as described below).
Venous gas emboli (VGE)	Gas bubbles in the venous circulation, typically used in reference to those bubbles detected with ultrasound post-decompression in veins or the venous heart chambers. Different scoring or grading scales are used for VGE quantification in vivo. VGE are often detected after scuba diving or rapid altitude exposure and are normally filtered by pulmonary capillaries without resulting in DCS. They may paradoxically enter the arterial circulation through arterio-venous shunting (cardiac or pulmonary), or if the lung filtering capacity is overwhelmed.

Decompression bubbles in the bloodstream are known as venous gas emboli (VGE). During return to the surface, VGE can form, grow, and coalesce [1, 3]. However, in some instances of decompression, VGE are not able to be fully removed from the bloodstream, causing the potential for critical blood flow to be impacted [3]. Impacts of decompression-induced VGE can vary from skin irritation to central nervous system impairment or stroke-like symptoms [1]. Post decompression VGE and subsequent DCS injury can be seen in small animal models subjected to extreme dive profiles [4–7]. Rodent models allow for translational experimentation that provides valuable information to the nature of these bubbles without requiring human subjects to conduct high risk dives.

Gas bubbles may be present in the venous or arterial vascular system, as well as in situ. However, presence of decompression bubbles will not necessarily result in decompression illness (DCI) [1]. Notably, VGE are often identified using ultrasound technology and have been shown to have some correlation to DCS outcome [8]. Ultrasound can detect bubbles due to their impedance mismatch with surrounding blood and tissues [5]. This characteristic has been used in diving research as a method for determining the presence of post-dive VGE. Ultrasonography tends to be the most popular imaging method in decompression bubble publications, which can likely be attributed to its real-time diagnostic capability and sensitivity to gas bubbles, as well as the fact that it is non-ionizing and highly portable [5, 6].

VGE detection has been applied to various animal models including rats and mice [4]. The presence of VGE can be described using one of several grading scales that have been previously developed (Kisman-Masurel or Spencer for Doppler, Eftedal-Brubakk for echocardiography) [7–9]. These scales produce a VGE grade based primarily on quantity of bubbles present per cardiac cycle. Previous work has shown that higher VGE grades correlate with an increased risk of DCS, and a VGE grade of zero corresponds with low risk for DCS [10, 11]. However, these VGE grades cannot be directly used as a surrogate endpoint for DCS outcome in dive trial studies aiming to test new decompression procedures [4]. Previous publications have used these scales to assess the risk associated with a certain dive profile, describe the impact of a certain treatment, and aid in tracking the onset of DCS in rats or mice [12–14].

**Fig 1:**
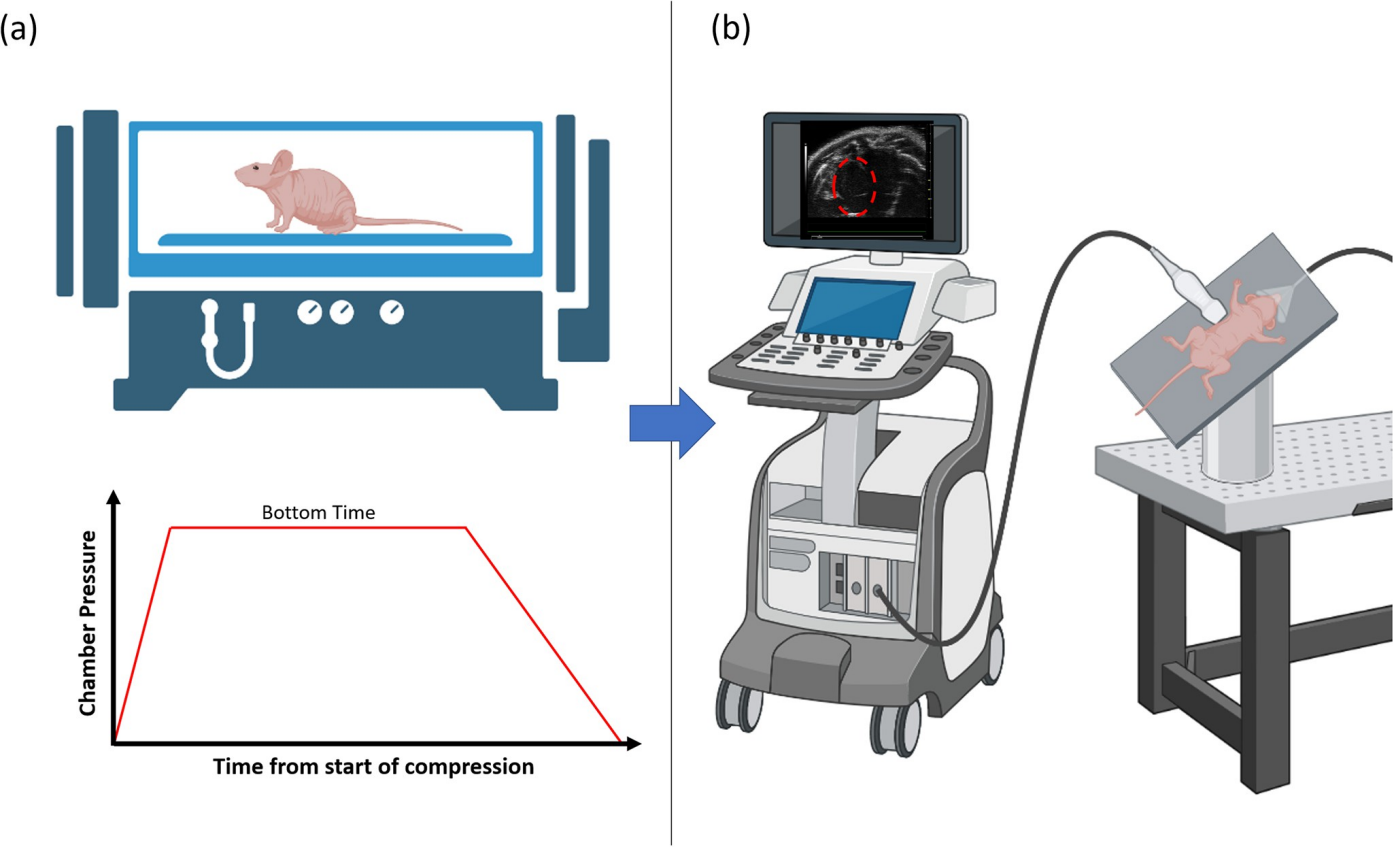
Schematic of rodent decompression experiment with bubble assessment. (a) A rodent is placed in a hyperbaric chamber (awake or anesthetized) and pressurized following a pre-determined pressure-time profile to simulate diving exposure (depicted in the bottom plot). (b) After decompression, the rodent is removed from chamber, anesthetized or restrained, and positioned for decompression bubble imaging. In this example, echocardiography is used to assess the presence of in vivo venous gas emboli (VGE) that may be seen in the venous heart chambers. The review will include bubble detection using other imaging modalities and encompass before, during and after diving timepoints.

Many decompression studies utilize small animal models to study decompression bubbles or VGE as additional biomarkers for DCS [6, 15–18]. Previous experiments have been conducted to assess rodent response to dives completed in a hyperbaric chamber using various non-invasive imaging modalities (Fig 1). However, there does not exist a comprehensive review that readily summarizes typical VGE imaging and analysis methods, thus it is important to identify and make available the results of previous work to provide researchers with the groundwork to develop standard guidelines for conducting reproducible rodent decompression experiments. Numerous experiments of this nature have been conducted over the last five decades and a scoping review is an appropriate process to properly synthesize and present relevant findings with regard to conducting similar experiments.

This scoping review will survey available rodent decompression publications that used ultrasound for VGE assessment and present a succinct review for future researchers. Results from this review will identify significant gaps in the field, while also reducing the need for duplicate in vivo experimentation, and be utilized for designing future experimentation. This information is anticipated to be useful for researchers in the decompression field that are studying rodents as a model for diving physiology or those using rodents for demonstration of new technologies prior to human translation [11, 14, 19–21]. Further, due to the size and physiological differences between humans and rodents, it is not always practical to apply the same methods for imaging these different subjects. Previous publications have applied common imaging practices and bubble analysis techniques used for humans, which may not provide ideal imaging data for assessing VGE in rodents. A comprehensive review is necessary to provide a roadmap of imaging methods used to assess rodents after decompression.

### Review questions

1. What are typical imaging modalities used for assessing decompression bubbles in rodents after a hyperbaric chamber dive?
2. What are the typical methods for assessing the bubble data collected by imaging?
3. What decompression protocols have been conducted with post-decompression bubble assessment?

## Materials and methods

This review will follow Joanna Brigg’s Institute Scoping Review Framework [22]. The scoping review process will involve keyword optimization, database identification and searches, a series of inclusion/exclusion rounds, and dissemination of results [22]. The Preferred Reporting Items for Systematic reviews and Meta Analyses extension for Scoping Reviews (PRISMA-ScR) checklist will be used to guide dissemination of results and any updates will be reflected in the completed version [23]. This protocol has been registered with the Open Science Framework (DOI: 10.17605/OSF.IO/TBFWN).

### Search methods

To maximize the collection of relevant sources, search terms will target three concepts: (1) Hyperbaric Experiments, (2) Rodent Model Species, and (3) Imaging Assessment (Table 2). Initial test searches were conducted to optimize the collected results. Using Boolean search operators AND/OR, selected databases will be explored for publications that fit all target search topics. The Elsevier interface will be used to implement the search strategy on the desired databases, where available. Relevant subject headings for each database will also be included (Table 2). Before finalization of our methods, the search strategy was peer-reviewed by a medical librarian, with experience using the Peer Review of Electronic Search Strategy [24].

**Table 2:** Search term framework.

	CONCEPT 1	CONCEPT 2	CONCEPT 3
**Keywords**	"decompression sickness*" OR "decompression illness*" OR dive OR diving OR bubbl* OR hyperbaric OR hypobaric OR "venous gas embol*" OR VGE OR "decompression trauma*"	imaging OR ultrasound* OR "magnetic resonance imag*" OR MRI* OR "CT scan*" OR "cat scan*" OR "compute* tomograph*" OR "x-ray*" OR xray* OR "anatomical scan*" OR "bubble detection" OR doppler*	mouse OR mice OR rat OR rats OR rodent* OR murine* or murinae

Search terms to be used in the review with Boolean search logic to target three concepts: (1) Decompression Experiments, (2) Non-Invasive Imaging Analysis, and (2) Rodent Model.

To bolster the search methods, the Medical Subject Headings (MeSH) were identified for each database (Table 3). The MeSH terms were found by searching each selected database for headings that contain, or are related to, any of the pre-determined keywords (Table 2).

**Table 3:** Selected subject headings framework.

Subject Headings			
**Pubmed**	Decompression Sickness/ Hyperbaric Oxygenation/ Embolism, Air/ Diving/	exp Magnetic Resonance Imaging/ Tomography, X-Ray Computed/ exp Ultrasonography, Doppler/	exp Mice/ exp Rats/ Murinae/
**Embase**	decompression sickness/ hyperbaric oxygen therapy/ gas embolism/ air embolism/ hyperbaric chamber/ hypobaric chamber/	exp nuclear magnetic resonance imaging/ computer assisted tomography/ exp doppler ultrasonography/ echocardiography/	exp mouse/ exp rat/ murine/
**Scopus**	[keywords only]	[keywords only]	[keywords only]

The selected search arrangement allows for publications to be collected based on keywords or search term relevance (Table 4). For databases that do not use MeSH, only keywords will be used to obtain results.

**Table 4:** Search methodology for PubMed.

Step	Search Definition (Keywords or MeSH)
1	"decompression sickness*" OR "decompression illness*" OR dive OR diving OR bubbl* OR hyperbaric OR hypobaric OR "venous gas embol*" OR VGE OR "decompression trauma*"
2	Decompression Sickness/ Diving/ Hyperbaric Oxygenation/ Embolism, air/
3	**1 or 2**
4	imaging OR ultrasound* OR "magnetic resonance imag*" OR MRI* OR "CT scan*" OR "cat scan*" OR "compute* tomograph*" OR "x-ray*" OR xray* OR "anatomical scan*" OR "bubble detection" OR doppler*
5	exp Magnetic Resonance Imaging/ Tomography, X-Ray Computed/ exp Ultrasonography, Doppler/
6	**4 or 5**
7	mouse OR mice OR rat OR rats OR rodent* OR murine* or murinae
8	exp Mice/ exp Rats/ Murinae/
9	**7 or 8**
10	**3 and 6 and 9**

### Database

We will search PubMed, Embase, and Scopus for relevant publications. If active at the time of the search, Rubicon Repository will also be included.

### Inclusion criteria

Publications will be included if the following Population, Concept, and Context points are satisfied:

#### Population

Rodents (rats and mice) that underwent a decompression exposure in a hyperbaric chamber.

#### Concept

Imaging methods used to visualize VGE or decompression bubbles in vivo during or after the decompression.

#### Context

Any publications, available in English, that match the target Population and Concept, without geographical or time restrictions.

Experiments that utilize ultrasound, magnetic resonance imaging, computed tomography, or X-ray imaging modalities to evaluate the specimen for decompression bubbles will be included. Publications will include all peer-reviewed journal articles, conference abstracts, or technical reports that are available in English and meet the inclusion criteria. Considering the number of decompression experiments conducted in the 1960’s-1980’s, time range will not be limited. Due to the limited size of the decompression field, exclusion criteria is limited to any publication that does not fit the pre-determined Population, Concept, and Context. For simplicity, only publications available in English will be considered.

### Study selection

**Fig 2:**
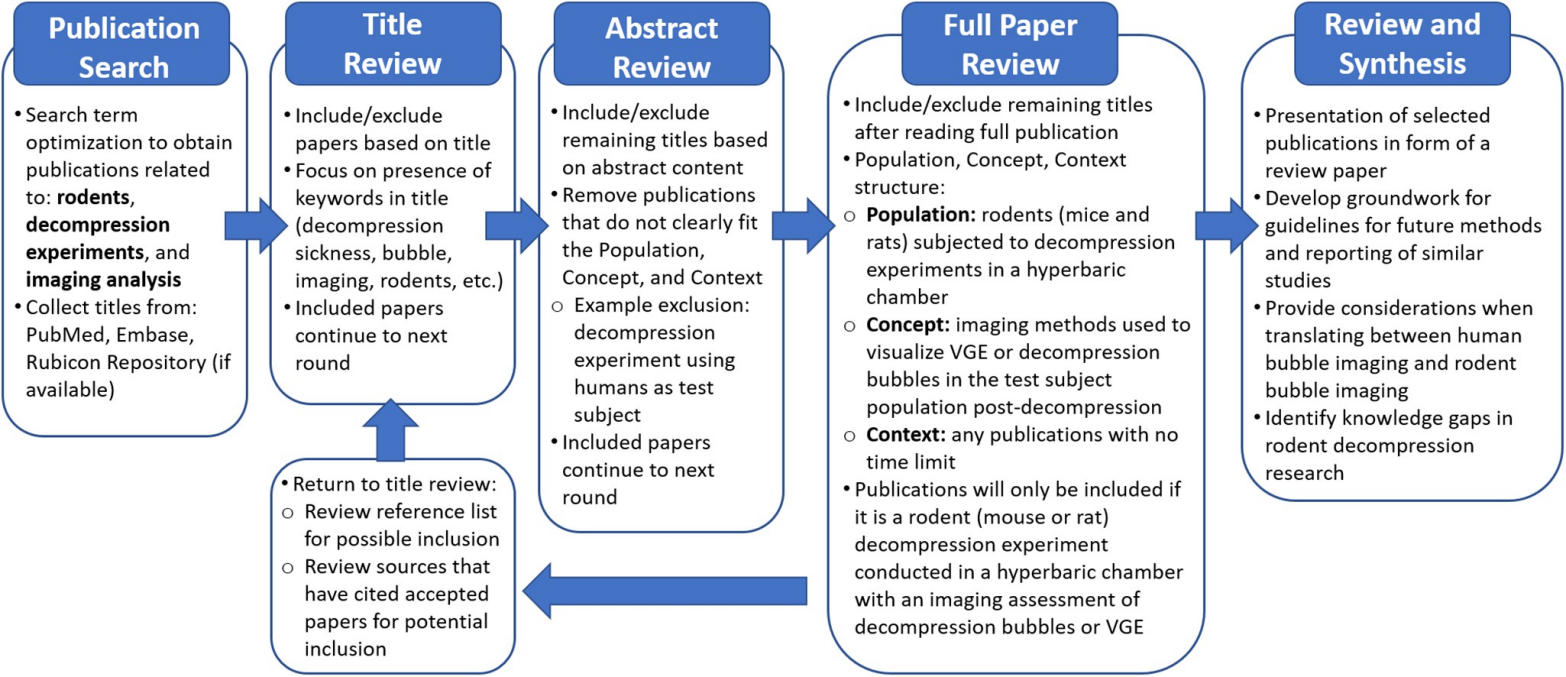
Publication review workflow. Relevant publications will be collected from preselected databases. Title selection includes multiple screening rounds based on relevancy of title, abstract, and a full-text read of the remaining titles.

Publication titles will be extracted from the selected databases and compiled into a master list, following methods presented in Fig 2. The master list will then be reduced in several truncating steps (Fig 2). First, duplicate titles will be removed within the citation manager. The remaining titles will be exported to the online Rayyan software [25]. Titles for publications will be reviewed, and an inclusion decision will be assigned to each publication based on its relevancy. Titles selected to be included will then be evaluated based on the abstract material. Screening steps will utilize the liberal accelerated method as described by Khangura et al., where the primary reviewer will complete all screening steps and will defer to a second reviewer for article exclusion [26]. This method requires two reviewers to exclude a title but only one reviewer to select an article for inclusion.

To counteract the limitations of focusing the search to PubMed, Embase, and Scopus databases, other relevant sources may be included through three alternative routes. First, any publications that are known to fit the search parameters can be nominated for inclusion, if the original search query does not include the title. Second, any publication that is cited by a title on the final source list may be considered for inclusion at the discretion of both reviewers. Third, articles that cite titles from the final list of included sources will be searched for. All three alternate inclusion options allow for sources that may not have been collected in the initial publication search, so long as the topic is relevant to the previously defined search parameters.

### Data extraction

After the full-text stage, each reviewer will independently complete a predetermined spreadsheet (Table 5). The extracted information will include relevant paper details including: first author, title, year published, and DOI. Other study specific information will also be collected including: rodent type, number of control animals, total number used, and study purpose. Decompression information is also desirable to compare between publications and extracted relevant information will include: dive pressure profile, dive information, and DCS outcomes (if applicable). The data extraction tool will be modified and revised as necessary throughout the review process.

**Table 5:** Data extraction template. Selected information to be extracted from each accepted paper during review process.

Desired Information	Description
Year of Publication	
First Author	
Link to Paper or DOI	
Type of Publication	Indicate if publication is a journal article, conference abstract, or other
Rodent Type	Indicate whether model specimen was rats or mice
Number of control animals	
Total number of animals included	
Number of raters	Indicate total number of raters that reviewed decompression bubble images
Bubble rating used	Indicate rating method used to grade decompression bubble images
Were the raters blinded?	Indicate whether raters were blinded to control and experimental groups
Study Purpose	Indicate goals and hypothesis for publication
Main finding of paper	Indicate if hypothesis was supported based on findings
Main finding of imaging	Indicate findings specifically relevant to imaging results
Imaging modality used	Indicate type of imaging modality used
Post-dive monitoring timepoints	Indicate total number of post-dive scans collected and the collection time with respect to the dive
Imaging settings	Describe settings and imaging system used for data collection
Dive profile and dive information	Describe specific details related to dive and other relevant information (for example: maximum depth, time at depth, breathing gas)
DCS outcome (if available)	Indicate rate of DCS occurrence and/or severity, including criteria used for diagnosis, if provided
Other information	

### Presenting results

Collected publications will be reviewed and a formative description of the completed work will be presented. This will not include quality appraisal or thematic analysis as these are not aspects of a scoping review [22]. Variation in data is expected throughout identified publications and will be reflected in the final review. The primary result of the review will be the completed data extraction template, with highlighted information from each publication included. The final review will include a frequency analysis that presents the number of publications reviewed that used each different imaging modality. The completed work will also include the finalized PRISMA-ScR checklist as a supplementary table to ensure reporting is in accordance with established guidelines [23]. Additionally, this review will lay the groundwork for a larger project to develop guidelines for minimum information to report in future rodent decompression bubble imaging publications. This will be done by highlighting information gaps to date and reporting of imaging parameters that could aid the reproducibility and comparison of similar experiments.

## Discussion

This review will present available information related to rodents, detectable decompression bubbles, and imaging methods that have been conducted in previous hyperbaric experiments. The resulting content analysis will include valuable information describing decompression bubble effects in rodents that have been characterized using non-invasive imaging. Future experimentation will likely benefit from this analysis by improving the visibility of previously completed rodent decompression experiments. This review aims to address multiple gaps in the field by providing guidelines for minimum information requirements for future publications, identify pitfalls when conducting experiments of this type, and discuss what is and is not possible with currently available methods.

### Limitations

This review focuses only on publications that included non-invasive imaging methods to assess rodents after decompression experiments. There are other articles in decompression research that may assess DCS using metrics such as; gait, skin irritation, or other symptoms. However, articles that do not include imaging for gas bubbles inside the test subject will not be included. Thus, this review will not broadly cover the entirety of rodent decompression experiments. Additionally, this review will only include publications available in English, which will exclude sources that have not been translated. This scoping review will also focus on publications available through the PubMed, Embase, and Scopus databases, plus reference lists and citations of included sources. We intend to review sources available through the Rubicon Foundation Repository due to its focus on decompression research. However, the site has been inaccessible due to maintenance during the time of preparing this protocol. In the event that Rubicon Repository is available prior to completion of this project, sources from this site will also be included in our scoping review.
